# Research Priority 3: ‘How can occupational therapists work more effectively with the family and carers of people who access services?’

**DOI:** 10.1177/03080226221135371

**Published:** 2022-11-03

**Authors:** Kerry Micklewright, Professor Morag Farquhar

**Affiliations:** 1 University of East Anglia and Norfolk, Norwich, Norfolk, UK; 2 Norwich University Hospital

Research is an essential pillar that supports our profession. It plays a crucial role in ensuring that we are providing evidence-based, cost-effective and impactful services that improve the lives of the individuals, groups and communities that rely upon them (RCOT, 2019). It is also instrumental in promoting innovation and helping us to adapt to future challenges. However, for research to most effectively support and shape current and future practice, it is important for us to appreciate that the relationship between research and practice should be reciprocal – that is, we should seek to understand not only the implications that research findings have for clinical practice, but also what unanswered research questions are most pressing from the perspectives of those that deliver and access services. In this way, we can ensure that we are funding and completing research that will have the greatest impact in terms of improving practice, promoting effective interventions and supporting those delivering and receiving our services.

The recent Occupational Therapy Priority Setting Partnership (OTPSP), wherein the Royal College of Occupational Therapists worked in partnership with healthcare professionals, carers, people who access occupational therapy services and the public to determine the top 10 research priorities for occupational therapy, provides valuable insight into where focused research could make the most difference (RCOT, 2021).

The third priority in this top 10 developed through the OTPSP asks how we can work more effectively with family members and carers. This is a particularly timely question given how deeply the COVID-19 pandemic affected the lives and highlighted the roles of informal carers – those unpaid family and friends who support people accessing our services. A concerning amount of evidence already suggested that providing care can be detrimental to the well-being of informal carers. When compared to the general population, they report poorer health, reduced happiness and increased loneliness, struggling with fatigue, stress, financial hardship and difficulties balancing caring responsibilities with employment (Carers UK, 2019; Foley et al., 2021; NHS, 2019). These are not a small minority of individuals; prior to the pandemic, approximately 6.5 million people – or 10% of the UK population – identified as informal carers for other adults (Carers UK, 2019). In addition to this, we must consider the informal carers of children accessing our services and the hidden, and often vulnerable, population of young carers, for whom caring responsibilities can impact on education, mental and physical well-being and social acceptance by their peers (Joseph et al., 2020). The COVID-19 pandemic resulted in approximately 4.5 million more informal carers, longer hours providing care, greater difficulty accessing services and formal support, and worsening physical and mental health for informal carers (Carers Week, 2020). There has long been evidence that services could, and should, be doing more to support them – and this is increasingly being emphasised in national policy (NHS, 2019). That this is reflected in one of our profession’s research priorities reinforces this message and challenges occupational therapists involved in research, practice, education and service management to consider how we could better work with, and meet the needs of, families and other informal carers.

The OTPSP advocates for investigation of how to ‘work more effectively’ with these individuals – and what this could look like may vary considerably depending on the area of practice and service structure. Occupational therapists work extremely closely with families and carers, utilising a flexible skillset to deliver a range of interventions to help support them in their roles, as evidenced by a recent review published in this journal (Micklewright and Farquhar, 2022). Whether providing direct support focused on the mental or physical well-being of a carer or family member, such as a relaxation group, or support to facilitate safer caring in the form of the advice, training and equipment, occupational therapists are well situated to make a real difference. However, the OTPSP makes clear that to fully realise the potential of occupational therapy to both better work with, and support, families and informal carers, we must embrace the need for further research to expand our evidence base and enable subsequent implementation of this in practice.

Aside from the benefits of this research to carers themselves – such as improvements in carer health and well-being – it is also important to consider that doing so could enable carers (if it is their wish) to sustain their caring role, benefitting those they care for in turn. Health and social care services rely on the informal support provided by families, friends, parents and young carers to reduce the size (and subsequent cost) of care packages, avoid hospital admissions, enabling individuals to live in their own homes (as opposed to expensive – and increasingly scarce – long-term residential or nursing placements) and deliver any suggested therapeutic inputs (such as exercises or environmental modifications) required in between direct sessions with therapists. The ways in which we work with carers can therefore influence the care and well-being of those they support, which adds an extra dimension to this research priority. It also potentially links this priority to others raised by the OTPSP, including exploration of how occupational therapy impacts on everyday lives (Priority 1), benefits individuals long term (Priority 4) and impacts on admissions avoidance (Priority 8); it additionally relates to our cost-effectiveness (Priority 10) given that support by informal carers reduces reliance on formal support.

It is clear that addressing how we work with carers is an important consideration for those delivering and accessing our services and, as such, this should be prioritised in our research going forwards. To help identify research that seeks to address this priority, we would suggest that this is clearly indicated in both the abstract and manuscript when submitting work for publication in this journal.
